# Hydrogen Sensors Using Nitride-Based Semiconductor Diodes: The Role of Metal/Semiconductor Interfaces

**DOI:** 10.3390/s110100674

**Published:** 2011-01-11

**Authors:** Yoshihiro Irokawa

**Affiliations:** National Institute for Materials Science, 1-1 Namiki, Tsukuba 305-0044, Japan; E-Mail: irokawa.yoshihiro@nims.go.jp; Tel.: +81-29-860-4549; Fax: +81-29-860-4914

**Keywords:** GaN, AlGaN, Schottky barrier diodes, hydrogen sensors

## Abstract

In this paper, I review my recent results in investigating hydrogen sensors using nitride-based semiconductor diodes, focusing on the interaction mechanism of hydrogen with the devices. Firstly, effects of interfacial modification in the devices on hydrogen detection sensitivity are discussed. Surface defects of GaN under Schottky electrodes do not play a critical role in hydrogen sensing characteristics. However, dielectric layers inserted in metal/semiconductor interfaces are found to cause dramatic changes in hydrogen sensing performance, implying that chemical selectivity to hydrogen could be realized. The capacitance-voltage (*C–V*) characteristics reveal that the work function change in the Schottky metal is not responsible mechanism for hydrogen sensitivity. The interface between the metal and the semiconductor plays a critical role in the interaction of hydrogen with semiconductor devises. Secondly, low-frequency *C–V* characterization is employed to investigate the interaction mechanism of hydrogen with diodes. As a result, it is suggested that the formation of a metal/semiconductor interfacial polarization could be attributed to hydrogen-related dipoles. In addition, using low-frequency *C–V* characterization leads to clear detection of 100 ppm hydrogen even at room temperature where it is hard to detect hydrogen by using conventional current-voltage (*I–V*) characterization, suggesting that low-frequency *C–V* method would be effective in detecting very low hydrogen concentrations.

## 1. Introduction

Recently, hydrogen has attracted considerable attention as a clean energy source instead of petroleum. Hydrogen also has many important applications such as its use in the processes of many industries that include the chemical, petroleum, food and semiconductor sectors. A hydrogen leak should be avoided because hydrogen when mixed with air in the ratio larger than ∼4 vol.% is explosive. For this reason, it has become very important to develop highly sensitive hydrogen detectors with a large variety of specifications to prevent accidents due to hydrogen leakage. Such detectors should allow continuous monitoring of the concentration of gases in the environment in a quantitative and selective way. Therefore, hydrogen sensors would form an integral part of any such systems incorporating hydrogen.

Solid-state hydrogen sensors under flow-through conditions are mainly classified into the following categories: pyroelectric, piezoelectric, fiber optic, electrochemical, and semiconductor sensors [1]. Among them, semiconductor device hydrogen detectors exhibit the highest sensitivity at elevated temperatures [1]. In many applications such as fuel cells and chemical process monitoring in industries, gas sensing with high sensitivity and low detection limit in harsh environments is required. Wide-band-gap compound semiconductor materials including SiC and GaN have been candidates for high-temperature gas sensing applications [2–5]. Due to the large band-gap of GaN and SiC, and the associated chemical stability and mechanical robustness, these semiconductors can be used for many harsh applications, satisfying a considerable interest in developing hydrogen gas sensors capable of operation in harsh environmental conditions such as high-temperature and chemically corrosive ambients. These include gas sensing operations during chemical reactor processing, onboard fire alarms on aircraft and space vehicles, as well as detection of fuel leaks in automobiles and aircraft, to name but a few. A unique advantage of a GaN gas sensor is that it can be integrated with GaN-based optical devices or high-power, high-temparature electronic devices on the same chip. Another advantage of nitride-based semiconductor devices is a utilization of AlGaN/GaN heterostructure. In an AlGaN/GaN heterostructure, the polarization-induced two dimensional electron gas (2DEG) concentration at the AlGaN/GaN interface is extremely sensitive to surface states. Any potential changes on the surface by adsorption of gas or liquid polar molecules, or pressure change would affect the surface potential and modulate the 2DEG density. Therefore, devices on AlGaN/GaN heterostructures have great potential for chemical gas sensing in harsh environments. Furthermore, the fabrication of nitride-based sensors on Si contributes to lower production cost. On the other hand, technical drawbacks for SiC devices are that the processing, particularly of SiC FETs (field-effect transistors), is inherently complicated, requiring high-temperature implantation and very high-temperature post-implantation annealing steps, leading to higher cost. Therefore, nitride-based semiconductor sensors are investigated in this report.

Hydrogen is reported to be able to alter the effective charge at the metal/semiconductor interface or metal/dielectric interface, resulting in changes in barrier height [6,7]. This effect is utilized in semiconductor based gas sensors fabricated on various semiconductor materials such as Si [8], SiC [4,5], and GaN [2,3]. The interaction of hydrogen with semiconductor devices has long been studied, and intensive research led to a model which attributes the reaction mechanism of the devices to hydrogen to the formation of a hydrogen-induced dipole layer at the metal/dielectric/semiconductor interface [8–12]. Lundström and co-workers investigated the influence of hydrogen on Pd or Pt–SiO_2_–Si structure using various methods, including internal photoemission, polarization currents, *C–V* measurements, and Kelvin probe. As a result, they concluded the interaction mechanism as follows: molecular hydrogen adsorbs on Pd or Pt surface and dissociates. Hydrogen atoms diffuse through Pd or Pt and adsorb at the metal–oxide interface, forming a dipole layer. The dipole layer is responsible for the work function change, for example, showing up as a voltage shift in the *C–V* characteristics of the device. Despite the existence of a considerable quantity of experimental data, however, there are still some debates as to the origin of the hydrogen sensitivity. For example, a work function decrease in the Schottky metals, such as Pd and Pt, on exposure to hydrogen is reported to be the origin of the changes in the characteristics of devices [13,14]. The role of the interface state density in the interaction of hydrogen with semiconductor devices is also discussed in previous reports [13]. Even now, the interaction mechanism of hydrogen with semiconductor devices still remains to be mysterious. In order to fabricate hydrogen sensors with higher performances, for example, those with selectivity for hydrogen, the interaction mechanism of hydrogen with semiconductor devices should be elucidated. Especially, the metal/semiconductor interfaces play a key role in the interaction mechanism in the devices. Here, I investigate the interaction mechanism of hydrogen with the nitride-based semiconductor diodes, focusing on the metal/semiconductor interfaces.

## 2. Experimental

Metal organic chemical vapor deposition (MOCVD) grown undoped GaN, Si-doped GaN (n-type 5 × 10^17^ cm^−3^) epilayers, and AlGaN/GaN heterostructures on (0001) Al_2_O_3_ substrates were used for this study, respectively. For Pt–GaN Schottky barrier diodes (SBDs), Ti(20 nm)/Al(100 nm)/Pt(40 nm)/Au(100 nm) multi-layers were formed on either 2 μm undoped GaN films or 2 μm Si-doped GaN films grown on undoped 1 μm GaN layers by lift off of electron beam evaporation as ohmic contacts. The contacts were subsequently annealed at 750 °C for 30 s under a flowing N_2_ ambient in a rapid thermal annealing (RTA) system. Then, Schottky contacts were formed by lift-off of electron beam deposited Pt(25 nm). For metal-insulator-semiconductor (MIS) Pt–GaN diodes, either a 10 nm SiO_2_ or a 10 nm Si_x_N_y_ dielectric layer was produced by radio-frequency sputtering at room temperature and lift-off before the Schottky contact formation. For Pt–AlGaN/GaN Schottky barrier diodes, the sample consisted of a buffer layer, an unintentionally doped GaN layer (3 μm thick), and an unintentionally doped AlGaN layer (20 nm) with an Al mole fraction of 24%. The AlGaN/GaN hetero-structure showed typical 2DEG properties with the sheet carrier concentration of 7.9 × 10^12^ cm^−2^ and the mobility of 1,237 cm^2^/Vs as determined by room-temperature Hall-effect measurements. After growth, planar SBDs were fabricated as follows. First, Ti/Al/Pt/Au metals were sequentially deposited and then sintered at 850 °C for 30 s to create ohmic contacts. Finally, 25 nm thick Pt films were formed as Schottky contacts. For all the fabricated devices, the diameter of circular Schottky contacts was 300 μm, and the distance between the ohmic contact and the Schottky metal was 20 μm.

Hydrogen interaction with the devices was investigated using a stainless-steel reaction chamber with tungsten probes under a flowing ambient (100 mL/min) of either N_2_ or H_2_ in N_2_ at a total pressure of 10.0 kPa at room temperature. Here, either 1% or 100 ppm H_2_ in N_2_ gas was employed. The *C–V* and conductance-voltage (*G–V*) characteristics were acquired using parallel circuit mode.

## 3. Results and Discussion

### 3.1. Effects of Interfacial Modification in the Devices on Hydrogen Detection Sensitivity

**Figure 1.**
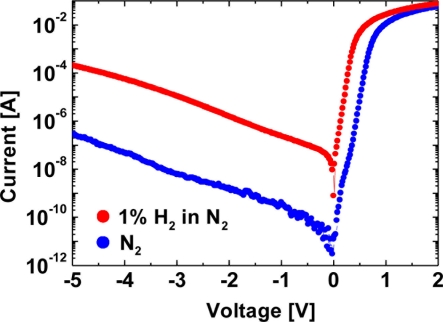
*I–V* characteristics for a Pt–(Si-doped) GaN Schottky diode in a N_2_ ambient and the response to the exposure to 1% H_2_ in N_2_ at room temperature. Reproduced with permission from reference [17]. Copyright The Japan Society of Applied Physics.

For GaN Schottky diode-type hydrogen sensors, it was reported that an oxidic intermediate layer between the catalytic Schottky contact and the GaN surface is the origin of the hydrogen sensitivity of Pd–GaN Schottky diodes, implying that the metal /semiconductor interfacial modification would lead to significant change in the interaction of hydrogen with devices [15]. Several authors have reported the critical role of the dielectric layer between the Schottky metal and the GaN surface in hydrogen sensitivity of devices [16–22]. In this report, firstly, hydrogen sensing characteristics of Pt–GaN Schottky diodes fabricated on intentionally defect-introduced GaN surfaces are investigated. Secondly, influence of dielectric layers, including SiO_2_, Si_x_N_y_ and AlGaN layers, between the metal and semiconductor interface on hydrogen detection was studied. Figure 1 shows the *I–V* characteristics for a Pt–(Si-doped) GaN Schottky diode in a N_2_ ambient and the response to the exposure to 1% H_2_ in N_2_ at room temperature.

The diode exhibits a good rectifying behavior in both ambients. The *I–V* characteristics consist of the exponential region in the low-current region and the resistance-limited region in the high-current region. On the basis of the thermionic emission model for *V* > 3*kT/q*, the *I–V* characteristics of Schottky diodes are described using:

(1) $$I_{F}={\mathrm{SA}}^{**}T^{2}\;\;\text{exp}(-\frac{q\Phi_{B}}{\mathrm{kT}})\left\{\text{exp}\left(\frac{\mathrm{qV}}{\mathrm{nkT}}\right)-1\right\}$$

where *I_F_* is the forward current, *S* is the diode area, A** is Richardson’s constant for n-GaN (24 Acm^−2^ K^−2^), *T* is the absolute temperature, *k* is Boltzmann’s constant, *q* is the electron charge, and *Φ_B_* is the Schottky barrier height [23]. The ideality factor *n* and Schottky barrier height *Φ_B_* are extracted from Equation (1). Table 1 shows a summary of the extracted *n* and *Φ_B_* in both N_2_ and 1% H_2_ in N_2_ from the *I–V* characteristics shown in Figure 1.

The obtained *n* and *Φ_B_* in N_2_ are consistent with those previously reported [24]. Upon exposure of the device to 1% H_2_ in N_2_ at room temperature, *n* and *Φ_B_* were found to be 1.22 and 0.662 eV, respectively. The reduction in the Schottky barrier height may be explained by the formation of a hydrogen-induced dipole layer between the metal and the semiconductor, which leads to the change in interfacial potential, as reported previously [6,7].

**Figure 2.**
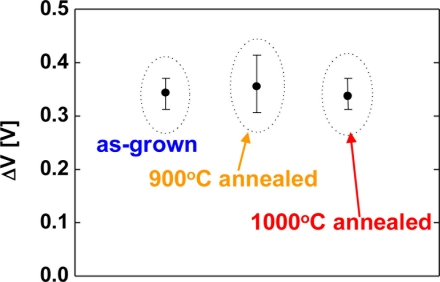
The sensitivity of Pt–(Si-doped) GaN Shottky diodes for 1% H_2_ in N_2_ at room temperature as a function of the annealing temperature of the GaN surfaces. The sensitivity is defined as a shift of voltage upon exposure to 1% H_2_ in N_2_ at room temperature at a current of 0.1 mA.

Next, hydrogen sensing characteristics of Pt–GaN Schottky diodes fabricated on as-grown and annealed GaN surfaces are studied in order to investigate the correlation between the surface defects of GaN and hydrogen sensing performance. The GaN samples were annealed before contact formation in the RTA system. Figure 2 shows the sensitivity of Pt–(Si-doped) GaN Schottky diodes for 1% H_2_ in N_2_ at room temperature as a function of the annealing temperature of the GaN surfaces. Here, the sensitivity is defined as a shift of voltage upon exposure to 1% H_2_ in N_2_ at a current of 0.1 mA. The error bars represent the range of the measured values.

**Figure 3.**
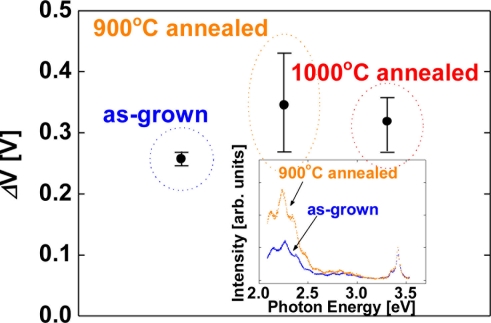
The sensitivity of Pt–(undoped) GaN Shottky diodes for 1% H_2_ in N_2_ at room temperature as a function of the annealing temperature of the GaN surfaces. The sensitivity is defined as a shift of voltage upon exposure to 1% H_2_ in N_2_ at room temperature at a current of 0.1 nA. The inset shows the room temperature photoluminescence spectra of as-grown and 900 °C annealed GaN surfaces.

As shown in Figure 2, the averaged value of the sensitivity of devices fabricated on as-grown surfaces, 900 °C and 1,000 °C annealed surfaces were 0.34, 0.35 and 0.34 eV, respectively. Significant differences of the sensitivity among the devices are not observed in Figure 2. In order to investigate the effects of carrier concentration of the films on hydrogen detection characteristics, undoped GaN films were employed. Figure 3 shows the sensitivity of Pt–(undoped) GaN Schottky diodes for 1% H_2_ in N_2_ at room temperature as a function of the annealing temperature of the GaN surfaces.

Here, the sensitivity is defined as a shift of voltage upon exposure to 1% H_2_ in N_2_ at a current of 0.1 nA. The error bars represent the range of the measured values. Although the devices fabricated on annealed surfaces displayed a slightly increased sensitivity, taking the error bars of the sensitivity into consideration, significant differences of the sensitivity among the devices are not observed, once again. On the other hand, the inset of Figure 3 shows the room temperature photoluminescence spectra of as-grown and 900 °C annealed GaN surfaces. The spectra have near-bandedge emission around 3.4 eV. The broad deep level emission around 2.2 eV is modulated by the Fabry-Perot interference fringes. As shown in the inset of Figure 3, 900 °C annealed GaN sample shows stronger deep level emission around 2.2 eV than as-grown sample does. This deep level emission is generally attributed to point defects and antisites, suggesting that high temperature annealing created these defects in the sample surface [25]. Though the PL spectra reveal the clear difference of the surface defects between as-grown and annealed surfaces as shown in the inset of Figure 3, the devices fabricated on annealed surfaces show the similar in hydrogen detection sensitivity to those on as-grown surfaces as shown in Figures 2 and 3. These results suggest that the surface defects of GaN do not play a critical role in hydrogen sensing characteristics of Pt–GaN Schottky diodes.

**Figure 4.**
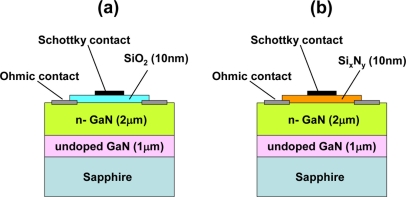
Schematic cross section of MIS Pt–GaN diodes with both 10 nm SiO_2_ **(a)** and 10 nm Si_x_N_y_ dielectrics **(b)**. Reproduced with permission from Reference [17]. Copyright The Japan Society of Applied Physics.

In order to investigate the influence of dielectric layers, including SiO_2_, Si_x_N_y_ and AlGaN layers, between the metal and semiconductor interface, on hydrogen detection sensitivity, hydrogen response of Pt–GaN diodes with dielectric layers was studied. Figure 4 shows the schematic cross sections of the MIS Pt–GaN diodes with both 10 nm SiO_2_ and 10 nm Si_x_N_y_ dielectrics, respectively.

**Figure 5.**
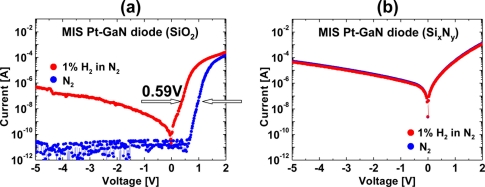
*I–V* characteristics of MIS Pt–GaN diodes with both 10 nm SiO_2_ **(a)** and 10 nm Si_x_N_y_ dielectrics **(b)** in N_2_ and response to exposure to 1% H_2_ in N_2_ at room temperature. Reproduced with permission from Reference [17]. Copyright The Japan Society of Applied Physics.

Figures 5(a,b) show the *I–V* characteristics of MIS Pt–GaN diodes with both 10 nm SiO_2_ and 10 nm Si_x_N_y_ dielectrics in N_2_ and response to exposure to 1% H_2_ in N_2_ at room temperature, respectively. As shown in Figure 5(a), hydrogen exposure of a MIS Pt–GaN diode with a SiO_2_ dielectric causes marked changes in *I–V* characteristics as compared with that of the conventional Schottky diode, as shown in Figure 1, demonstrating that the MIS devices with a SiO_2_ dielectric have a higher sensitivity to hydrogen than the conventional Schottky devices. Table 2 shows the sensitivities of the conventional Pt–GaN Schottky diode and MIS Pt–GaN diodes in 1% H_2_ in N_2_ extracted from Figures 1 and 5. Here, the sensitivities are defined as shifts in voltage upon exposure to 1% H_2_ in N_2_ at a current of 0.1 mA.

As shown in Table 2, the MIS Pt–GaN diode with a 10 nm SiO_2_ dielectric shows a marked improvement in hydrogen detection sensitivity, which is twice higher than that of the conventional Pt–GaN Schottky diode. Upon changing the hydrogen-containing ambient into N_2_, the *I–V* characteristics of the MIS Pt–GaN diode are found to revert to the initial values, although it takes a long time, that is, more than three hours at room temperature, just like the conventional Pt–GaN Schottky diode. In sharp contrast, a MIS Pt–GaN diode with a 10 nm Si_x_N_y_ dielectric does not show any hydrogen response, as shown in Figure 5(b). Therefore, the hydrogen detection sensitivity of the device is found to be 0.00 V, as shown in Table 2. More detailed electrical properties of these MIS Pt–GaN diodes upon exposure to hydrogen are discussed in Section 3.2.

**Figure 6.**
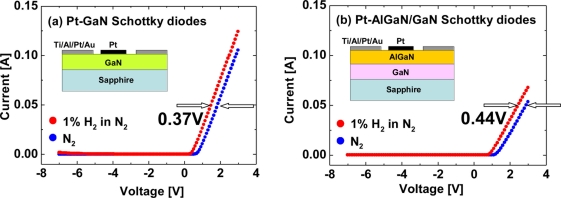
*I–V* characteristics of Pt–GaN diode **(a)** and Pt–AlGaN/GaN diode **(b)** in N_2_ and response to exposure to 1% H_2_ in N_2_ at room temperature.

Hydrogen sensing characteristics of Pt–AlGaN/GaN diodes are generally reported like those of Pt–GaN diodes. In Pt–AlGaN/GaN diodes, the AlGaN/GaN hetero-interface structurally plays a major role in carrier transportation in an SBD. Moreover, in an AlGaN/GaN hetero-structure, the polarization-induced 2DEG concentration at the AlGaN/GaN interface is extremely sensitive to surface states. Any potential changes on the surface by adsorption of gas or liquid polar molecules, or pressure change would affect the surface potential and modulate the 2DEG density, leading to application in excellent sensors. Since the AlGaN layer is undoped and considered to be an insulator, the AlGaN/GaN structure would be regarded as one of MIS structures. Previously, only a few studies revealed the investigation of hydrogen detection sensitivity of Pt–GaN and Pt–AlGaN/GaN Schottky diodes [26], and little is known about the comparative performances. In this report, hydrogen detection sensitivity of Pt–GaN and Pt–AlGaN/GaN Schottky diodes is compared, and the results are shown in Figure 6.

As shown in Figure 6, the sensitivities of the Pt–GaN and Pt–AlGaN/GaN diodes are 0.37 V and 0.44 V, respectively. Here, the sensitivities are defined as shifts in voltage upon exposure to 1% H_2_ in N_2_ at a current of 0.05 A. The Pt–AlGaN/GaN diode shows slightly higher sensitivity as compared with that of the Pt–GaN device, consistent with the previous report [26]. Note that the Pt–GaN diode show relatively higher leakage current in H_2_ ambient, as shown in Figure 6(a). In sharp contrast, Pt–AlGaN/GaN diode shows no leakage current in both ambients. Therefore, Pt–AlGaN/GaN devices would be suitable for application to highly-sensitive hydrogen sensors operated at high temperature. The detailed hydrogen sensing mechanism of Pt–AlGaN diodes are discussed in Section 3.3.

### 3.2. Hydrogen-Induced Change in the Electrical Properties of Metal-Insulator-Semiconductor Pt–GaN Diodes

In the previous section, I compared the hydrogen response of MIS Pt–GaN diodes with both 10 nm SiO_2_ and 10 nm Si_x_N_y_ dielectrics. As a result, MIS Pt–GaN diodes with a SiO_2_ dielectric showed a marked improvement in hydrogen detection sensitivity. In sharp contrast, MIS Pt–GaN diodes with a Si_x_N_y_ dielectric did not show any hydrogen response. Since the changes in the *I–V* characteristics of devices are totally different for the two dielectric layers, detailed investigation for these phenomena would lead to the elucidation of the interaction mechanism of hydrogen with semiconductor devices. Here, *I–V* and *C–V* characteristics are performed for MIS Pt–GaN diodes with both SiO_2_ and Si_x_N_y_ dielectrics, and the changes in the electrical properties are discussed.

**Figure 7.**
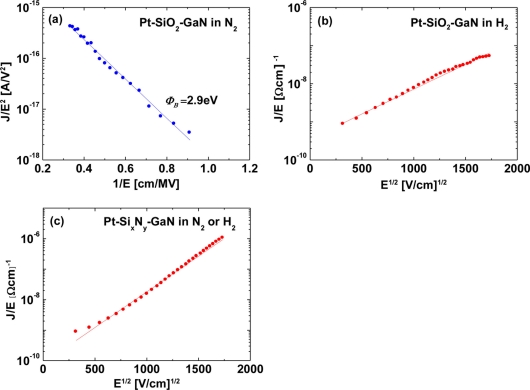
**(a)** FN plot for MIS Pt–GaN diodes with a SiO_2_ dielectric in a N_2_ ambient. **(b)** Pool–Frenkel plot for MIS Pt–GaN diodes with a SiO_2_ dielectric in a H_2_ ambient. **(c)** Pool–Frenkel plot for MIS Pt–GaN diodes with a Si_x_N_y_ dielectric in a N_2_ ambient Reproduced with permission from Reference [22]. Copyright 2010, American Institute of Physics.

The conduction mechanism for MIS Pt–GaN diodes with both SiO_2_ and Si_x_N_y_ dielectrics is investigated as follows. In the oxide field range of 0.3–0.9 MV/cm, for MIS Pt–GaN diodes with a SiO_2_ dielectric, the measured *I–V* data in a N_2_ ambient were fitted using the Fowler–Nordheim (FN) tunneling model, as shown in Figure 7(a).

The FN model is given by:

(2) $$J={\mathrm{AE}}^{2}\;\text{exp}(-\frac{B}{E})$$

(3) $$A=\frac{q^{3}m}{8\pi {\mathrm{hm}}_{\mathrm{dielec}}\Phi_{B}}$$

(4) $$B=\frac{8\pi \sqrt{2m_{\mathrm{dielec}}}{\left(q\Phi_{B}\right)}^{3/2}}{3\mathrm{qh}}$$

where *m_dielec_* is the effective electron mass in the dielectric, *m* is the free electron mass, *q* is the magnitude of electron charge, *Φ_B_* is the barrier height, and *E* is the dielectric electric field [27]. Here, the value of the effective mass used was equal to *m_dielec_* = 0.5 *m* [28]. Since the plot of log (*J*/*E^2^*) *versus* 1/*E* is linear, as shown in Figure 7(a), the oxide conduction is FN conduction. The barrier height extracted from the FN plot as shown in Figure 7(a) is 2.9 eV, and this value is found to be consistent with the previously reported values for the SiO_2_ /GaN interface [28,29].

On the other hand, for MIS Pt–GaN diodes with a SiO_2_ dielectric, the measured *I–V* data in a H_2_ ambient were fitted using the Pool–Frenkel emission model, as shown in Figure 7(b). The Pool–Frenkel emission model is given by:

(5) $$J\propto E\;\text{exp}(-\frac{q(\phi_{B}-\sqrt{\mathrm{qE}/\pi \varepsilon })}{\mathrm{kT}})$$

where *E* is the dielectric electric field, *ε* is the dielectric permittivity, *ϕ_B_* is the trap level below the conduction band, *T* is the temperature, *k* is the Boltzmann’s constant [30]. The plot of log (*J*/*E*) *versus E^1/2^* is linear, as shown in Figure 7(b), and, therefore, the oxide conduction is Pool–Frenkel emission. It is found that the hydrogen ambient drastically changes the conduction mechanism of MIS Pt–GaN diodes with a SiO_2_ dielectric, as shown in Figures 7(a,b). For MIS Pt–GaN diodes with a Si_x_N_y_ dielectric, the measured *I–V* data in a N_2_ or H_2_ ambient were fitted using the Pool–Frenkel emission model, as shown in Figure 7(c). Figure 7(c) shows a linear relation, suggesting that the dielectric conduction is Pool–Frenkel emission. The Si_x_N_y_ dielectric is well known for showing Pool–Frenkel emission [28,31,32], consistent with the result shown in Figure 7(c). The Pool–Frenkel emission is due to emission of trapped electrons into the conduction band. The supply of electrons from the traps is through thermal excitation. For trap states with Coulomb potentials, the expression is similar to that of the Schottky emission. The barrier height, however, is the depth of the trap potential well. The barrier reduction is larger than in the case of Schottky emission by a factor of two, since the barrier lowering is twice as large due to the immobility of the positive charge [33]. The principal defect in the band gap of Si_x_N_y_ was reported to be the silicon dangling bond [31]. The hydrogen ambient does not change the conduction mechanism for MIS Pt–GaN diodes with a Si_x_N_y_ dielectric, as shown in Figure 5(b).

**Figure 8.**
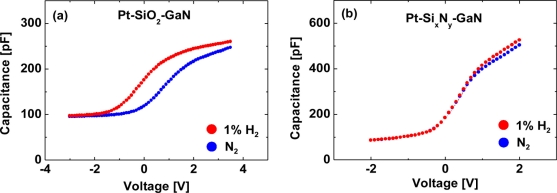
Room-temperature *C–V* characteristics at 10 kHz of the MIS Pt–GaN diodes with both SiO_2_ **(a)** and Si_x_N_y_ **(b)** dielectrics in a N_2_ ambient and the response to the exposure to 1% H_2_ in N_2_. Reproduced with permission from Reference [22]. Copyright 2010, American Institute of Physics.

Figures 8(a,b) show room-temperature *C–V* characteristics at 10 kHz of the MIS Pt–GaN diodes with both SiO_2_ and Si_x_N_y_ dielectrics in a N_2_ ambient and the response to the exposure to 1% H_2_ in N_2_, respectively. Bias voltage was swept from accumulation to depletion.

Note that the capacitances in the accumulation region for MIS Pt–GaN diodes with a Si_x_N_y_ dielectric is about twice as large as those for MIS Pt–GaN diodes with a SiO_2_ dielectric, consistent with their dielectric constants that are 3.8–4.2 for SiO_2_ and 7.5 for Si_x_N_y_, respectively. As shown in Figure 8(a), for MIS Pt–GaN diodes with a SiO_2_ dielectric, the *C–V* curve in H_2_ significantly shifts toward negative bias values. The capacitance at each value of bias is just shifted by the difference of a constant voltage so that the shape of the *C–V* curve is unaltered. These *C–V* curves are usually explained by hydrogen-induced dipole layer formed in the metal–dielectric interface [12]. That is, the atomic hydrogen formed on the Pt surface diffuses through the metal film and is trapped at the metal-insulator interface where a polarized layer is formed. The hydrogen response *ΔV* can be expressed as:

(6) $$\Delta V=\frac{n_{i}\mu }{\varepsilon }$$

where *n_i_* is the concentration of hydrogen atoms at the interface, *μ* is the effective dipole moment per trapped hydrogen atom, *ε* is the permittivity [34]. The hydrogen-induced shift in the *C–V* curves, *ΔV*, for MIS Pt–GaN diodes with a SiO_2_ dielectric is 1.0 V, as shown in Figure 8(a). This value is much larger than the *C–V* curve shift of 0.3 V for Pt–AlGaN/GaN Schottky barrier diodes at the similar experimental condition, [as shown in Figure 12(a)], reflecting the drastic *I–V* changes in MIS Pt–GaN diodes with a SiO_2_ dielectric. Based on the Equation (6), the values of *n_i_* and *μ* should depend on the properties of interfaces. In other words, hydrogen could be a probe in order to investigate the interface properties of the devices. The *C–V* curves at a measurement frequency of 1 MHz display the similar value of shift upon exposure to hydrogen.

In sharp contrast, for MIS Pt–GaN diodes with a Si_x_N_y_ dielectric, the *C–V* curve does not show any shifts upon exposure to hydrogen. Note that the capacitances in the accumulation region (the voltages ranging from 1 to 2 V) in H_2_ show slight increase as compared with those in N_2_. This is probably due to the change in the dielectric constant of Si_x_N_y_ in the different ambients. The results shown in Figure 8(b) reveal that the hydrogen-induced dipole layer is not formed in the metal–dielectric interface for MIS Pt–GaN diodes with a Si_x_N_y_ dielectric. These *C–V* characteristics are quite anomalous and have not been reported yet. There is a key point that emerges from the results shown in Figure 8. Previously, it is reported that a work function decrease in the Schottky metals, such as Pd and Pt, on exposure to hydrogen is the origin of the hydrogen sensitivity, that is, changes in the characteristics of devices [13,14]. If the proposed mechanism is true, the results shown in Figure 8 is not consistent, because no work function change is observed in Figure 8(b), despite using the same Schottky metal in the both devices. Note that the work function change should be reflected in the flatband voltage shifts in the *C–V* curves. Therefore, it is obvious that the interface between the metal and the semiconductor plays a critical role in the interaction of hydrogen with semiconductor devices.

**Figure 9.**
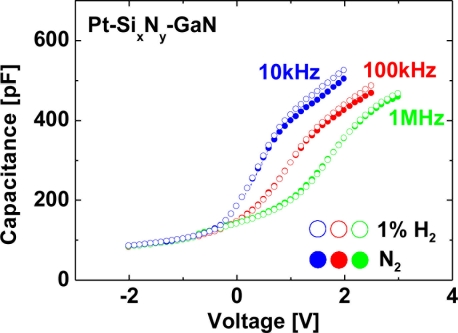
Room-temperature *C–V* characteristics of the MIS Pt–GaN diodes with a Si_x_N_y_ dielectric at 10 kHz, 100 kHz and 1 MHz in a N_2_ ambient and the response to the exposure to 1% H_2_ in N_2_.

Figure 9 shows room-temperature *C–V* characteristics of the MIS Pt–GaN diodes with a Si_x_N_y_ dielectric at measurement frequencies of 10 kHz, 100 kHz and 1 MHz in a N_2_ ambient and the response to the exposure to 1% H_2_ in N_2_.

As shown in Figure 9, the *C–V* curves for MIS Pt–GaN diodes with a Si_x_N_y_ dielectric show frequency dispersion at measurement frequencies ranging from 10 kHz to 1 MHz. Although the *C–V* curves for MIS Pt–GaN diodes with a Si_x_N_y_ dielectric show frequency dispersion at measurement frequencies ranging from 10 kHz to 1 MHz, all the *C–V* curves do not show any shifts upon the exposure to hydrogen regardless of measurement frequencies. Note that these *C–V* characteristics of the MIS Pt–GaN diodes with both SiO_2_ and Si_x_N_y_ dielectrics in H_2_ shown in Figure 8 are found to revert to the initial values upon changing the hydrogen-containing ambient into N_2_, just like the *I–V* characteristics.

Although the detailed reaction mechanism of these MIS Pt–GaN diodes to hydrogen is unknown at present, the plausible mechanisms are described as follows. First, the hydrogen-induced changes in the electrical properties may be related to the number of adsorption sites for hydrogen at the metal/dielectric/semiconductor interface of the devices [35]. Second, hydrogen could be contained in both the SiO_2_ and Si_x_N_y_ dielectrics during the sputtering deposition, and some of the hydrogen molecules trapped at the metal/dielectric interface may affect the hydrogen detection sensitivity. Third, the Si_x_N_y_ is well known to have a high density of trap states and hence potentially suffer from charge trapping instabilities [32]. This property of the Si_x_N_y_ may relate to the acquired *I–V* and *C–V* data. Otherwise, these obtained results may be explained only by a novel reaction mechanism. Therefore, it would be important to understand the reaction mechanism underlying these phenomena, possibly leading to chemical selectivity to hydrogen. Especially, more detailed investigation of the interface states in a Schottky contact could help to interpret the reaction mechanisms at the interfaces of the MIS Pt–GaN diodes to hydrogen.

Furthermore, Si_x_N_y_ passivation is known to be effective in improving the reliability of AlGaN/GaN high-electron mobility transistors (HEMTs), and the stability of MIS Pt–GaN diodes with a Si_x_N_y_ dielectric exposed to hydrogen, as reported here, may be related to the excellent passivation property of Si_x_N_y_ [36].

### 3.3. Low-Frequency Capacitance-Voltage Study of Hydrogen Interaction with Pt–AlGaN/GaN Schottky Barrier Diodes

**Figure 10.**
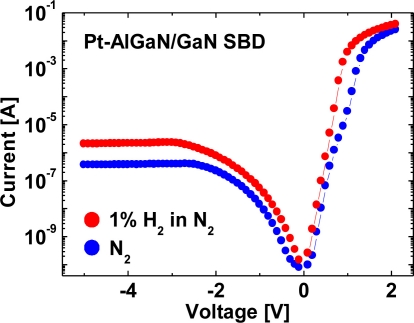
*I–V* characteristics of the device in N_2_ and response to exposure to 1% H_2_ in N_2_ at room temperature. Reproduced with permission from Reference [37]. Copyright Wiley-VCH Verlag GmbH & Co. KGaA.

In the previous studies, high-frequency *C–V* characterization was typically employed in order to elucidate the reaction mechanism. As a result, a shift of the *C–V* curves toward negative bias values upon hydrogen exposure was observed, concluding that the formation of a dipole layer reduces the effective Schottky barrier height. The formation of the dipole layer should increase the capacitance; however, no increase of the capacitance has been reported yet. We presumed that very low frequencies are required in order to observe the proposed dipole layer and to investigate the reaction mechanism. Here, we demonstrate a clear change in the *C–V* characteristics of Pt–AlGaN/GaN SBDs exposed to hydrogen using a low frequency [37]. Typical *I–V* characteristics of the Pt–AlGaN/GaN SBD in N_2_ and response to exposure to 1% H_2_ in N_2_ at room temperature are shown in Figure 10.

From *I–V* measurements, good rectifier characteristics of the n-type Schottky diode were confirmed for SBD samples in both environments. The introduction of hydrogen clearly enhances the current under a given bias both in forward and reverse *I–V* characteristics, as reported previously [38,39]. The forward *I–V* characteristics consist of the exponential region in the low-current region and the resistance-limited region in the high-current region. The ideality factor *n* and Schottky barrier height *Ö_B_* were calculated by applying the thermoionic emission theory to the forward *I–V* curves. The calculated values of *n* and *Ö_B_* were 2.3 eV, 0.88 eV in N_2_ and 1.9 eV, 0.81 eV in 1% H_2_, respectively. These values are consistent with those previously reported [39]. Upon changing the hydrogen-containing ambient into N_2_, the *I–V* characteristics of the Pt–AlGaN/GaN SBD are found to revert to the initial values. According to the previous literature, the reduction in the Schottky barrier height is explained as a result of the formation of a hydrogen-induced dipole layer between the metal and the semiconductor [38,39].

**Figure 11.**
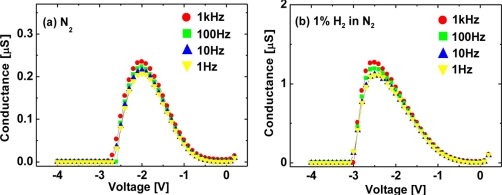
Room-temperature *G–V* characteristics at various frequencies of the Pt–AlGaN/GaN SBD in N_2_ **(a)** and response to exposure to 1% H_2_ in N_2_ **(b)**.

**Figure 12.**
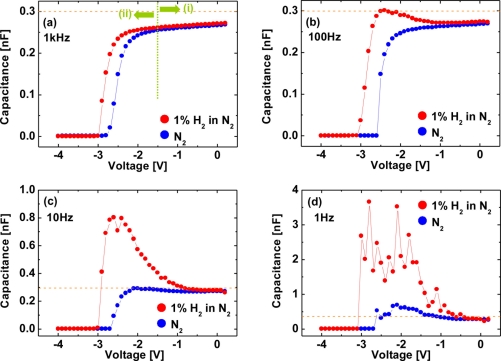
*C–V* characteristics at 1 kHz **(a)**, 100 Hz **(b)**, 10 Hz **(c)** and 1 Hz **(d)** of the Pt–AlGaN/GaN SBD in N_2_ and response to the exposure to 1% H_2_ in N_2_ at room temperature. Reproduced with permission from Reference [37]. Copyright Wiley-VCH Verlag GmbH & Co. KGaA.

In order to investigate the detailed mechanism of the interaction between hydrogen and Pt–AlGaN/GaN SBDs, we have conducted low frequency *G–V* and *C–V* characterization of Pt–AlGaN/GaN SBDs exposed to hydrogen. The *G–V* and *C–V* characterization was conducted with an ac modulation level of 100 mV and frequencies ranging from 1 Hz to 1 kHz. Figures 11 and 12 show room-temperature *G–V* and *C–V* characteristics at various frequencies of the Pt–AlGaN/GaN SBD in N_2_ and response to exposure to 1% H_2_ in N_2_, respectively.

The *G–V* curves in Figure 11 suggest the following three issues: First, the conductance goes through a peak and approaches zero on either side. Second, the maximum values of the conductance in H_2_ is much higher than those in N_2_, which is probably related to the enhanced current under a given bias in *I–V* characteristics upon the introduction of hydrogen, as shown in Figure 10. Third, the *G–V* curves show frequency dependence. The peak magnitude of the conductance increases with increasing frequency in both ambients.

In Figure 12(a), the *C–V* characteristics in N_2_ can be roughly classified into two diode bias (*V_G_*) regions: (i) −1.5 V ≤ *V_G_*, (ii) *V_G_* < −1.5 V. In region (i), the capacitance is almost flat due to only small changes induced by applying *V_G_*, where the depletion layer width is calculated to be 22.2–23.3 nm, in reasonable agreement with the AlGaN layer thickness. The almost flat portion of the capacitance is interpreted as the dc depletion of 2DEG by negative dc voltages. In region (ii), the capacitance rapidly decreases with decreasing *V_G_* down to −2.8 V which is the pinch-off voltage, *V_TH_*. This region corresponds to the depletion of GaN after almost complete pinch-off of 2DEG. At a frequency of 1 kHz, the *C–V* curve in H_2_ shifts toward negative bias values, as reported previously [38]. As the frequency decreases from 1 kHz to 1 Hz, the capacitance in H_2_ dramatically increases and the oscillation of the capacitance is observed, as shown in Figure 12. These *C–V* characteristics are quite anomalous and have not been reported so far. In sharp contrast, the *C–V* curves in N_2_ do not change significantly over all the frequencies ranging from 1 kHz to 1 Hz. Note that these *C–V* characteristics of the Pt–AlGaN/GaN SBD in H_2_ shown in Figure 12 are found to revert to the initial values upon changing the hydrogen containing ambient into N_2_, just like the *I–V* characteristics. There are several key points that emerge from the results shown in Figure 12. First, at a frequency of 1 kHz, the *C–V* curve in H_2_ does not reflect any polarization because the capacitance should increase when polarization is formed in the dielectric material [40]. In Figure 12(a), the capacitance at each value of bias is just shifted by the difference of their pinch-off voltages so that the shape of the *C–V* curve is unaltered. These *C–V* curves could be explained by positive fixed charge in the Pt–AlGaN interface [41]. Second, at a frequency of 1 Hz, the capacitance in H_2_ dramatically increases with decreasing *V_G_* down to *V_TH_*. This anomalous *C–V* curve would not be attributed to mobile ionic charge because mobile ionic charge can be detected in *C–V* characteristics even at much higher frequency like 1 MHz [41]. Third, the capacitance in region (ii), e.g., at a bias voltage of −2 V, at a frequency of 1 Hz in N_2_ is slightly higher than that at a frequency of 1 kHz. This is probably due to the tiny amount of remnant hydrogen in the devices. Here, no thermal treatment was performed in order to expel the remnant hydrogen from the devices prior to the experiment. Fourth, generally speaking, interfacial polarization, which occurs when mobile charge carriers are impeded by a physical barrier that inhibits charge migration, could produce such an increased capacitance in H_2_ at a low frequency [40]. Although little is known about interfacial polarization in the case of hydrogen interaction with semiconductor devices, hydrogen-related dipoles may have the function of interfacial polarization. Adsorbed hydrogen on the AlGaN surface is positively charged and fixed on the surface, forming a dipole layer. Sufficiently low ac oscillations switch the polarity of dipoles, leading to the significant increase in the capacitance. Fifth, as for the *C–V* curves at low frequencies, the capacitance in H_2_ dramatically increases with decreasing *V_G_* down to *V_TH_* as compared with those in N_2_ although the capacitance at zero bias has nearly the same value both in N_2_ and H_2_, suggesting that the alignment of electric dipoles by applied biases may be related. A similar phenomenon is observed in ferroelectric materials in which the domains can switch from one direction of spontaneous alignment to another when an electric filed is applied, giving rise to large changes in the polarization and dielectric constant [42]. Furthermore, the oscillation of the capacitance observed in the *C–V* curve at 1 Hz in H_2_ may be explained by the alignment of hydrogen-related dipoles when the ac oscillation is applied, *i.e.*, the ac oscillation tries to switch the polarity of dipoles, but some of them cannot follow the oscillation simultaneously, resulting in the capacitance oscillation as a function of the applied bias voltages. In addition, surface charges on AlGaN may influence the dipole formation and alter *C–V* curves, implying that low-frequency *C–V* measurements could be useful for monitoring the surface states of AlGaN.

**Figure 13.**
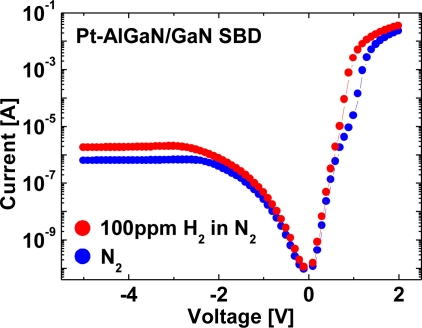
*I–V* characteristics of the device in N_2_ and response to exposure to 100 ppm H_2_ in N_2_ at room temperature.

*I–V* and *C–V* characteristics of the Pt–AlGaN/GaN SBD for much lower concentration of hydrogen, which was 100 ppm H_2_ in N_2_, were performed at room temperature. Figure 13 shows *I–V* characteristics of the device in N_2_ and response to exposure to 100 ppm H_2_ in N_2_ at room temperature. Comparing Figure 13 with Figure 10, the current enhancement under a given bias is different, *i.e.*, 1% H_2_ enhances more current under a given bias than 100 ppm H_2_ does both in forward and reverse *I–V* characteristics.

**Figure 14.**
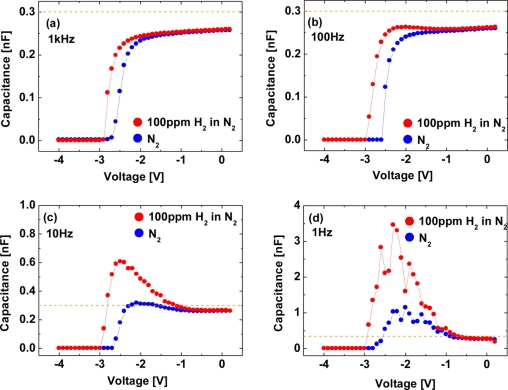
*C–V* characteristics at 1 kHz **(a)**, 100 Hz **(b)**, 10 Hz **(c)** and 1 Hz **(d)** of the Pt–AlGaN/GaN SBD in N_2_ and response to exposure to 100 ppm H_2_ in N_2_ at room temperature.

**Figure 15.**
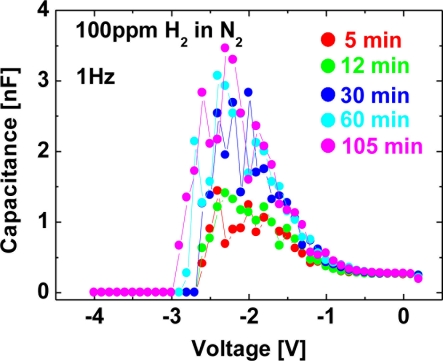
Time evolution of *C–V* characteristics in 100 ppm H_2_ at 1 Hz.

Figure 14 shows *C–V* characteristics at 1 kHz (a), 100 Hz (b), 10 Hz (c) and 1 Hz (d) of the Pt–AlGaN/GaN SBD in N_2_ and response to exposure to 100 ppm H_2_ in N_2_ at room temperature, and Figure 15 shows time evolution of *C–V* characteristics in 100 ppm H_2_ at 1 Hz.

Note that the *C–V* characteristics with 100 ppm H_2_ result in similar curves to those with 1% H_2_, as shown in Figures 12 and 14, but the saturation time for 100 ppm H_2_ was much longer than that for 1% H_2_. As shown in Figure 15, upon H_2_ exposure, the capacitances increase till ∼60 min and saturate after 60 min upon H_2_ exposure.

By sharp contrast, at 1% H_2_, capacitances saturate instantaneously. In low H_2_ concentrations like 100 ppm, it takes more than 60 min to saturate the *C–V* characteristics (and also *I–V* characteristics), as shown in Figure 15, suggesting that hydrogen-induced dipole formation needs longer time in lower concentration of a hydrogen ambient. But, using low-frequency *C–V* characterization leads to clear detection of 100 ppm hydrogen even at room temperature where it is hard to detect hydrogen by using conventional *I–V* characterization, suggesting that low-frequency *C–V* method would be effective in detecting very low concentration of hydrogen.

## 4. Conclusions and Future Work

The hydrogen sensing mechanism of nitride-based semiconductor diodes is investigated, focusing on the metal/semiconductor interfaces. Electrical characterization of Pt–GaN Schottky diodes shows the devices fabricated on annealed surfaces display the similar in hydrogen detection sensitivity to those on as-grown surfaces unlike the difference of the PL spectra, implying that the surface defects of GaN do not play a critical role in hydrogen sensing characteristics of Pt–GaN Schottky diodes. However, dielectrics between the metal and GaN surfaces are found to cause dramatic change of hydrogen detection performance, suggesting that the realization of chemical selectivity to hydrogen is possible.

Exposure of Pt–SiO_2_–GaN diodes to hydrogen at room temperature is found to change the conduction mechanisms from FN tunneling to Pool–Frenkel emission. In sharp contrast, Pt–Si_x_N_y_–GaN diodes exhibit Pool–Frenkel emission in nitrogen and do not show any change in the conduction mechanism upon exposure to hydrogen. The *C–V* curve for Pt–Si_x_N_y_–GaN diodes also does not show any shifts upon the exposure to hydrogen. Although the detailed reaction mechanism of these MIS Pt–GaN diodes to hydrogen is unknown at present, these results suggest that the work function change in the Schottky metal is not responsible mechanism for the hydrogen sensitivity. The interface between the metal and semiconductor plays a critical role in the interaction of hydrogen with semiconductor devices. Detailed investigation for these phenomena would lead to the elucidation of the interaction mechanism of hydrogen with semiconductor devices. Furthermore, hydrogen could be a probe in order to investigate the interface properties of the devices.

A significant increase in the capacitance was observed at a low frequency in Pt–AlGaN/GaN SBD upon exposure to hydrogen. This could be explained by interfacial polarization which is attributable to hydrogen related dipoles, *i.e.*, sufficiently low ac oscillations switch the polarity of dipoles, leading to an increase in the capacitance. In addition, using low-frequency *C–V* characterization leads to clear detection of 100 ppm hydrogen even at room temperature where it is hard to detect hydrogen by using conventional *I–V* characterization, suggesting that low-frequency *C–V* method would be effective in detecting very low concentration of hydrogen.

Future work will involve the following three points: First, more detailed interaction mechanism of hydrogen with nitride-based semiconductor devices should be studied. In this report, only the interaction of hydrogen with nitride-based semiconductor diodes at room temperature was investigated by using electrical methods. Investigation varying the gases, measurement temperatures, device structures and measurement methods may lead to the more specific elucidation of the interaction mechanism [43,44]. Second, fabrication of sensors with higher performances is required. The following list gives both constraints and requirements for an ideal detector: (i) Chemically selective, (ii) reversible, (iii) fast, (iv) highly sensitive, (v) durable, and (vi) non-contaminating and non-poisoning; other constraints involve requirements for (vii) simple operation, (viii) small size (portability), (ix) simple fabrication, (x) relative temperature insensitivity, and (xi) low noise. For example, no literatures previously report semiconductor sensors with selectivity to hydrogen. The elucidation of interaction mechanism of hydrogen with semiconductor devices may lead to realization of the selectivity. Third, from the point of view of the interaction mechanism of hydrogen with semiconductor materials and devices, relation with other semiconductor materials, such as ZnO, SiC and diamond should be clarified. For example, adsorbed hydrogen on ZnO, SiC or diamond is found to change their electrical properties drastically [45–47]. Similar phenomenon may be observed in nitride-based semiconductors, possibly leading to a new interaction mechanism of hydrogen with semiconductors.

## Figures and Tables

**Table 1.** Extracted *n* and *Φ_B_* in both N_2_ and 1% H_2_ in N_2_ from the *I–V* characteristics shown in Figure 1. Reproduced with permission from Reference [17]. Copyright The Japan Society of Applied Physics.

	**N_2_**	**1% H_2_**
Ideality factor, n	1.30	1.22
Schottky barrier height (eV)	0.838	0.662

**Table 2.** Sensitivities of conventional Pt–GaN Schottky and MIS Pt–GaN diodes in 1% H_2_ in N_2_ extracted from Figures 1 and 5. Here, the sensitivities are defined as shifts in voltage upon exposure to 1% H_2_ in N_2_ at a current of 0.1 μA. Reproduced with permission from Reference [17]. Copyright The Japan Society of Applied Physics.

	**Sensitivity (V)**
Schottky diode	0.30
MIS diode (SiO_2_)	0.59
MIS diode (Si_x_N_y_)	0.00
